# Randomized trial evaluating an mHealth intervention for the early community-based detection and follow-up of cutaneous leishmaniasis in rural Colombia

**DOI:** 10.1371/journal.pntd.0011180

**Published:** 2023-03-27

**Authors:** Mabel Castillo, Neal Alexander, Luisa Rubiano, Carlos Rojas, Andrés Navarro, Domiciano Rincon, Leonardo Vargas Bernal, Yenifer Orobio Lerma, Nancy Gore Saravia, Eliah Aronoff-Spencer

**Affiliations:** 1 Centro Internacional de Entrenamiento e Investigaciones Médicas, CIDEIM, Cali, Colombia; 2 Universidad Icesi, Cali, Colombia; 3 Facultad Nacional de Salud Pública, Universidad de Antioquia, Medellín, Colombia; 4 Grupo i2t, Universidad Icesi, Cali, Colombia; 5 University of California, San Diego, California, United States of America; Hebrew University-Hadassah Medical School, ISRAEL

## Abstract

**Background:**

In Latin America, cutaneous leishmaniasis primarily affects dispersed rural communities, that have limited access to the public health system and medical attention. Mobile health (mHealth) strategies have shown potential to improve clinical management and epidemiological surveillance of neglected tropical diseases, particularly those of the skin.

**Methods:**

The Guaral +ST app for Android was designed to monitor cutaneous leishmaniasis treatment and assess therapeutic response. We carried out a randomized trial in the coastal municipality of Tumaco in southwestern Colombia, with parallel arms comparing a) follow-up aided by the app to b) standard institution-based follow-up. Treatment was prescribed according to national guidelines. Follow-up of therapeutic response was scheduled at the end of treatment and at 7, 13 and 26 weeks after the start of treatment. The primary endpoint was the proportion of participants who were monitored at or around week 26, allowing outcome and effectiveness of treatment to be determined.

**Results:**

Follow-up of treatment and outcome assessment was achieved in significantly more patients in the intervention arm than the controls, Of the 75 participants in the two randomized arms, 74 had information on whether or not treatment was followed and outcome determined at or around week 26. Among these, 26/49 (53.1%) were evaluated in the intervention arm, and none (0/25, 0%) in the control arm (difference = 53.1%, 95% confidence interval 39.1–67.0%, p<0.001). Of the 26 participants evaluated at or around week 26 in the intervention arm, 22 (84.6%) had cured. There were no serious adverse events, nor events of severe intensity among patients monitored by CHW using the app.

**Conclusion:**

This study provides proof of concept for mHealth to monitor treatment of CL in remote and complex settings, deliver improved care and to provide information to the health system on the effectiveness of treatment as it is delivered to affected populations.

**Clinical Trial Registration:**

**ISRCTN54865992**.

## Author summary

Cutaneous leishmaniasis is a parasitic disease which causes sores on the skin than can spread to other skin or mucosal locations and lead to permanent scaring and disfigurement. In Latin America, cutaneous leishmaniasis primarily affects remote rural communities with limited health services. Mobile health (mHealth) strategies, such as mobile phone apps, have potential to improve care and surveillance of cutaneous leishmaniasis and other skin diseases. We developed a mobile phone app called Guaral +ST for use by community health volunteers to monitor cutaneous leishmaniasis treatment. We carried out a randomized trial in the coastal municipality of Tumaco in southwestern Colombia. Patients with cutaneous leishmaniasis were treated in accordance with national guidelines, and randomly allocated to either follow-up aided by the app, or to standard institution-based follow-up. Of the 74 randomized participants with the necessary data, 26/49 (53.1%) were evaluated in the intervention arm, and none (0/25, 0%) in the control arm. Hence follow-up was achieved more frequently by community health volunteers supported by the mHealth app than conventional institutional-based monitoring of treatment. This study shows that mHealth apps can help deliver improved care of cutaneous leishmaniasis in remote settings.

## Introduction

Cutaneous leishmaniasis (CL) is emblematic of “neglected tropical diseases” (NTDs), a group of chronic infections that affect the world’s poorest citizens and remain largely uncontrolled [1]. Throughout Latin America, cutaneous leishmaniasis primarily affects inhabitants of dispersed rural communities where occupational intrusion into the sylvatic cycle, and adaptation of sand fly vectors to peridomiciliary settings promote transmission. Lesions typically occur on skin sites that are exposed to sandfly bites, such as the face, arms and legs, and the resulting scars can cause stigmatization, anxiety, and depressive symptoms [2]. Approximately 0.7–1.2 million CL cases occur each year, with around 75% of these being in only ten countries [3]. One of these countries is Colombia, which reports the highest number of cases among the Andean countries, and is second only to Brazil among the endemic countries of Latin America [4].

Rural communities have limited access to the public health system and medical attention, contributing to delayed diagnosis, increased morbidity, and attrition in adherence to prescribed treatment. Data on effectiveness and adverse events of treatment administered within routine care, are unavailable in Colombia and several countries of Latin America [4]. Innovative mobile health (mHealth) strategies have shown potential to improve clinical management and epidemiological surveillance of skin neglected tropical diseases [5–10] and other infectious diseases in resource limited settings. Our recent work has focused on the development of mHealth applications (apps) for NTDs through the lens of CL diagnosis and monitoring of treatment. This study builds upon prior work to evaluate the performance of an mHealth intervention that was co-designed with community health volunteers (CHV) and subsequently used by them. We report the findings and lessons of a randomized trial of the mHealth-facilitated monitoring of treatment and its outcome by community health volunteers.

## Methods

### Ethical approval

The trial protocol was approved on 29 August 2016 by the Comité Institucional de Ética de Investigación en Humanos (Institutional Ethics Committee for Research Involving Humans) of CIDEIM (reference 08–2016). The study was monitored by this CIDEIM Institutional Review Board. Voluntary, informed, signed consent was provided by each participant.

### Design of the mHealth app and of the randomized trial

**Fig 1:**
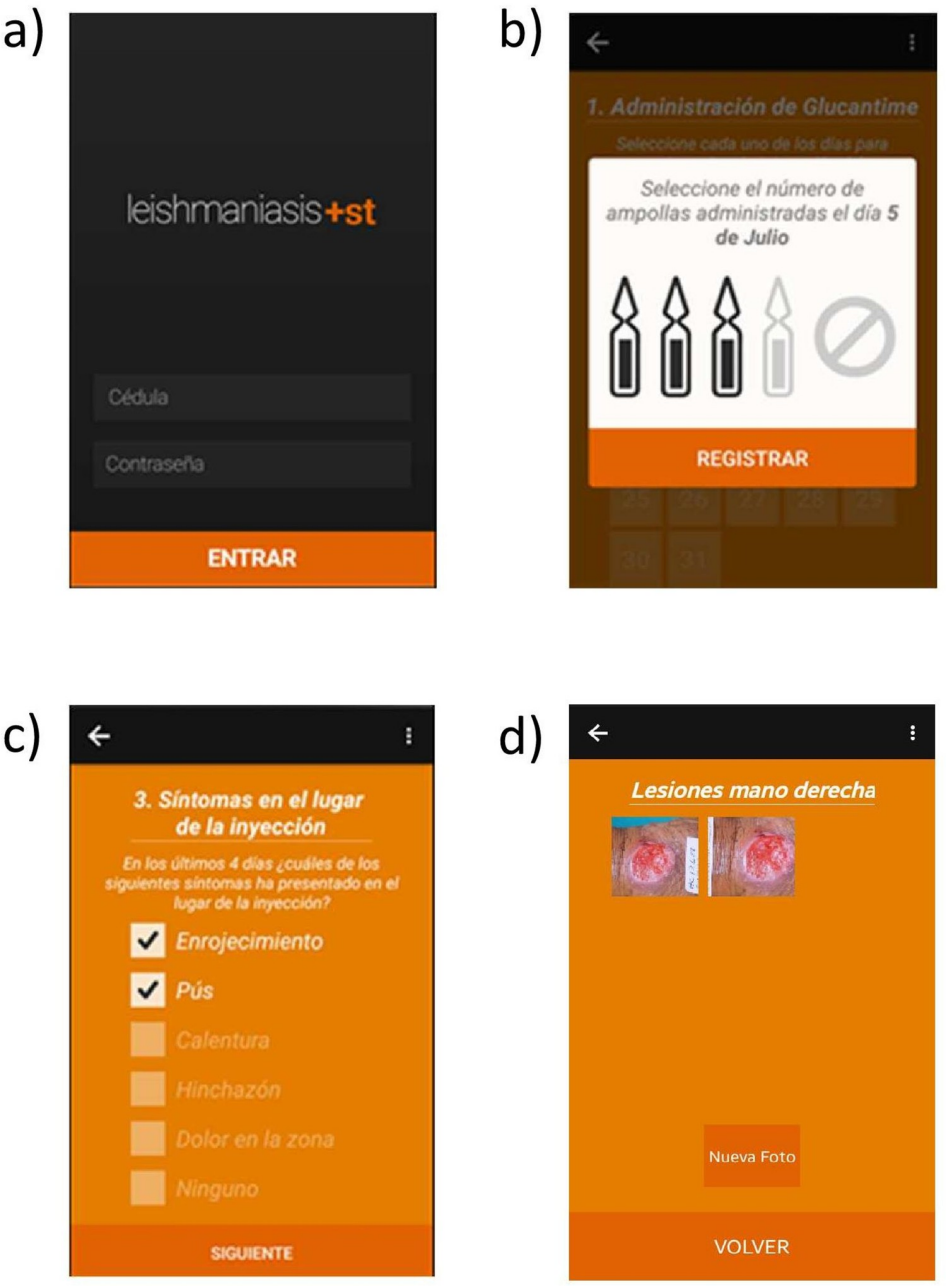
Guaral +ST app. a) Initial screen of the app. b) Screen for registering the administered dose of treatment. c) Screen for registering injection site reactions. d) Lesion photographs registered via the app.

The Guaral +ST app for Android is designed to monitor cutaneous leishmaniasis treatment and assess therapeutic response [11]. It has three main functions: i) promoting adherence to pharmacologic treatment, ii) measurement of adverse events associated with treatment, via periodic follow-up, and iii) assessment of therapeutic response via photographic evaluation. This evaluation strategy has been found to be sensitive and specific, relative to in-person assessment [12]. The intended users are community health volunteers and other healthcare workers. Fig 1 shows screenshots of the app.

We conducted a randomized trial to evaluate the performance of the app in the hands of CHV. We did not hypothesize that the intervention would improve outcomes of the initial treatment but rather that it would result in more complete data on treatment outcomes and adverse events. The trial had parallel arms comparing a) follow-up by community health volunteers aided by the app to b) standard institution-based follow-up. A non-randomized control group was added later (see below). The trial, including its protocol, was retrospectively registered with ISRCTN, reference number ISRCTN54865992 (https://doi.org/10.1186/ISRCTN54865992).

### Setting

**Fig 2:**
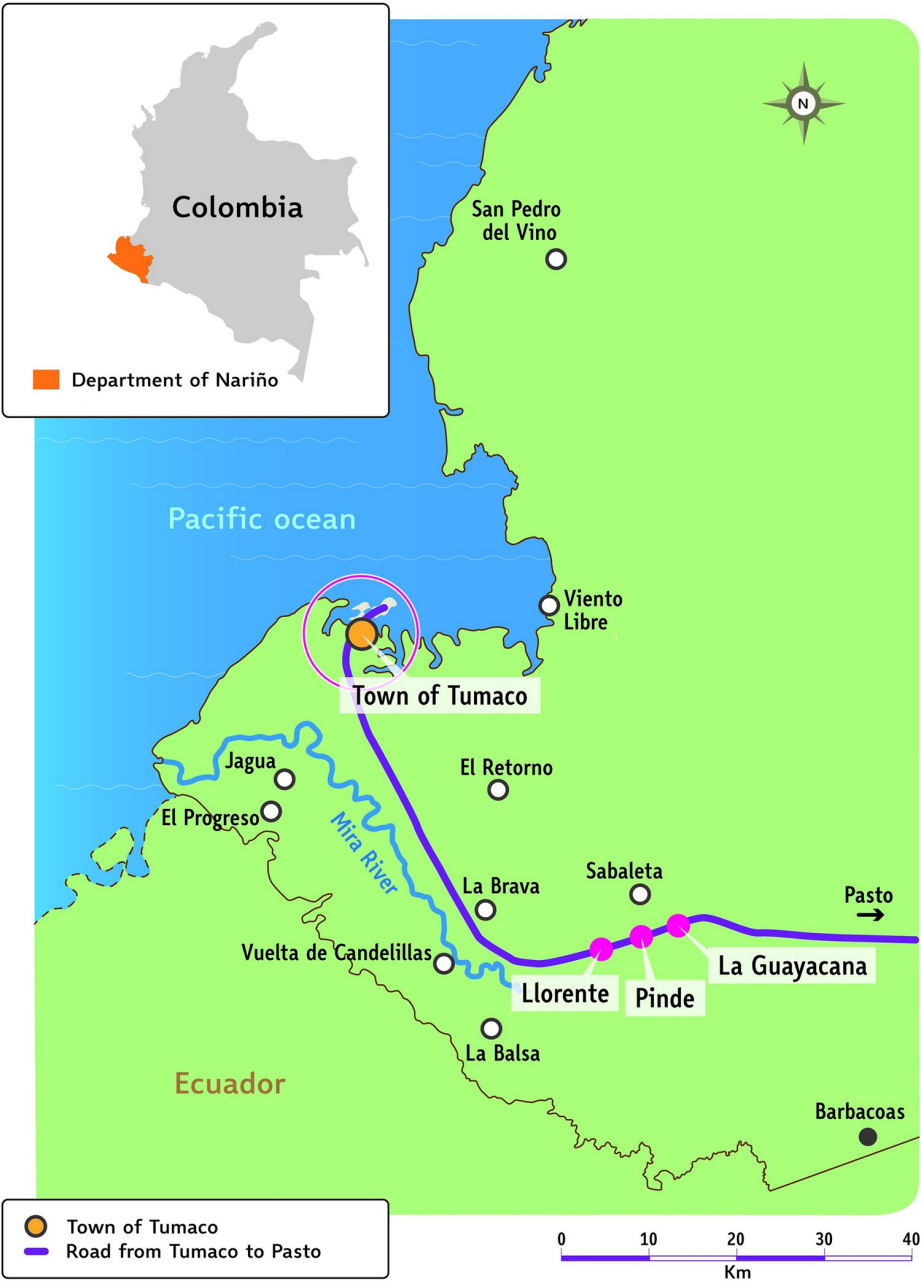
Study area. Tumaco municipality. The CIDEIM facility is in Tumaco town. During the study, participants had to reside in, or have access to, one of the three *veredas* shown as pink dots. Other names are of selected places of permanent residence of study participants. The international boundary is available from the GADM database of global administrative areas (https://gadm.org/download_country.html) under the licence shown at https://gadm.org/license.html. Locations were approximated from a map made by the United Nations Office for the Coordination of Humanitarian Affairs [26]. All are in Tumaco municipality except one (Barbacoas) shown as a solid black dot.

The coastal municipality of Tumaco borders Ecuador in southwestern Colombia, in the Department of Nariño, and comprises 365 townships (*veredas*) (Fig 2). Armed conflict has contributed to internal population displacement. One paved road links the main urban center, also referred to as Tumaco, to the Departmental capital, Pasto, which is in the Andes mountains. Otherwise, travel is by unpaved roads or, more frequently, by river or sea on boats. Hence, travel to the town of Tumaco, where the health centers are located, may take up to 8 hours, which restricts access to health services. The use of mHealth tools by health volunteers offers the opportunity to overcome these barriers. CIDEIM maintains a health service point of care in the Tumaco urban centre.

### Evaluators

Six Community Health Volunteers (CHV) were selected from among inhabitants of the rural study area. The CHV had experience in the use of mobile phones, and some had participated in training in the use of the Guaral app for presumptive diagnosis application [5]. For the current study, they received training in cutaneous leishmaniasis, its pharmacological treatment, follow-up of therapeutic response, use of smart phones, photographic recording of lesions with the mobile phone camera, and the Guaral +ST application. The CIDEIM health service team was also trained to support the CHV in the field work.

### Screening, diagnosis and treatment

Patients made between 3 and 6 visits to the CIDEIM health service. Visits 0 and 1 were for diagnostic confirmation and treatment formulation, respectively (Table 1). Those randomized to the intervention (app) arm received additional home visits by a CHV. More specifically, at Visit 0, patients were assessed using a clinical prediction rule (CPR) implemented in the Guaral app. Presumptive cases were considered as potential participants of the current trial, and a physical examination was then performed. Those with active lesions suggestive of leishmaniasis were evaluated using routine parasitological methods (tissue smear or, if negative, culture of lesion aspirate, see "Parasitological assays" below). Patients with confirmed CL disease received one of the treatments recommended by the Colombian Ministry of Social Protection [13]: meglumine antimoniate or miltefosine, for 20 or 28 days respectively.

**Table 1:** Baseline characteristics of study participants (n = 104).

	App intervention (n = 49)	Randomized control (n = 26)	Non-randomized control (n = 29)
**Age** (years): median (range)	26 (2–78)	24.5 (3–62)	28 (6–70)
**Sex**: n (%)			
Female	18 (36.7)	5 (19.2)	9 (31.0)
Male	31 (63.3)	21 (80.8)	20 (69.0)
**Ethnicity**: n (%)			
Afrocolombian	26 (53.1)	15 (57.7)	15 (51.7)
Indigenous	9 (18.4)	5 (19.2)	6 (20.7)
*Mestizo*	13 (26.5)	6 (23.1)	7 (24.1)
Missing	1 (2.0)	0 (0)	1 (3.4)
**Weight** (kg): median (range)	65 (13–96)	65 (12–88)	63 (18–96)
**Number of active CL lesions**: median (range)	2 (1–7)	1 (1–8)	2 (1–5)
**Time from symptom onset to parasitological diagnosis**: days median (range)	59 (9–310)	67.5 (6–683)	43 (0–143)

### Inclusion and exclusion criteria

Inclusion criteria:

- Residence in, or access to, one of the three *veredas* previously identified as a) having relatively high incidence of CL in the national surveillance system [14], b) being safe for work to be conducted, and c) coverage by a Community Health Volunteer and bidirectional accessibility (i.e. by both patients and CHV). The locations of these three *veredas* are shown in Fig 2.
- Any age.
- At least one parasitologically confirmed cutaneous lesion.
- Any gender.
- Any ethnicity.
- Acceptance of the informed consent process and signature of the form.

Exclusion criteria:

- Mucosal leishmaniasis.
- A condition that required treatment in the health center rather than the area of residence.

Although not excluded by the protocol, in practice it was not possible to include pregnant or breastfeeding women, because the drugs recommended for first line use for CL are contraindicated in these groups.

### Randomization

If a CHV was available within a feasible distance of the patient’s residence, the patient was randomized between the two arms. The sequence was previously prepared by a statistician who did not have patient contact. Variable size blocks were used, specifically of length 2, 4, 6, and 8, generated by the blockrand() function of R version 3.3.1, resulting in a sequence with 50 and 25 records for the intervention and control groups respectively, that is, a 2:1 ratio [15, 16]. This sequence was accessed from Tumaco via CIDEIM’s web application for patient enrollment. The physician and principal investigator only learned of each patient’s arm allocation after enrollment, i.e. the allocation was concealed. Following enrollment neither the investigators nor the patients were blinded to the allocation.

### Non-randomized control group

Eligible patients could not be randomized if a CHV was not available near their residence. The study was expanded in order to enroll such patients and hence increase study recruitment. The first patient in this non-randomized control group was enrolled in January 2017. The procedures for such patients were the same as those in the randomized control arm.

### Follow-up during treatment

In the case of patients who received any part of their treatment at the CIDEIM health facility in the town of Tumaco, monitoring was carried out by CHV, liaising with the health facility. For those patients who resided in villages where there was no CHV to supervise the treatment, monitoring was coordinated with CHV from other communities. In addition to the app, a standard paper form was also used by CHV to monitor treatment adherence and adverse events for all study participants, as is done for all leishmaniasis patients in CIDEIM. In addition, for those in the app arm, weekly monitoring of treatment was performed via the mobile application (Guaral+ST), recording the number of doses received and the adverse events, including their severity and frequency. At each visit, i.e. prior to treatment, at the end of the treatment, and at weeks 7,13, and 26, a photograph of each lesion was taken with the mobile phone by CHVs. Study procedures are summarized in S1 Table. The trial is reported in accordance with the CONSORT guidelines (S2 Table).

### Endpoints

Adherence was calculated as the percentage of the prescribed doses that were taken by the patient. For meglumine antimoniate this is expressed in terms of ampoules. The primary endpoint was the proportion of participants who were monitored at or around week 26, which is within the time range for the consensus definition of final cure [17]. We refer to this timing as target study week 26 because not all such visits occurred at exactly week 26. For target study weeks 7,13 y 26, a window of ± 2 weeks was allowed, and ±1 week for the end of treatment visit.

### Sample size

The number in the intervention group was planned to be double that in the control group [16]. Although the target power was 80%, the sample size was capped at 75 for feasibility. Based on previous studies [18], we assumed a proportion of 35% followed up at week 26 in the intervention arm, and 8% in the control arm. The significance level was set at 5% significance level (two-sided), and the power was 75%, as estimated from “power two proportions” in Stata [19].

### Adverse events

In the intervention (app) arm, each week CHV recorded the symptoms, severity, and frequency of adverse events that occurred during treatment. These were reported to the study physician for guidance on clinical management, e.g. in determining whether to stop treatment. The app allowed CHV to monitor the clinical characteristics and evolution of the lesions, and to take and transmit photographs. In the control groups, the patient recorded any adverse events on a printed checklist that was delivered to the health personnel of the clinical facility.

### Therapeutic response

In the intervention (app) arm, the study physician evaluated the therapeutic response via photographs taken by the CHV using the phone app at four time points. In-person assessment by the study physician was only undertaken if the app photos were not clear or if it was required for patient management, e.g. for adverse events or concomitant medication. Evaluation of clinical response was performed at the end of treatment, and at target study weeks 7, 13 and 26 after starting treatment, whether in-person or via assessment of lesion photographs taken with the Guaral +ST app by the CHV. Due to a technical error, the exact dates of visits were not retained in the app dataset, but rather only as the target study week.

In the control (standard of care) groups, evaluation of therapeutic response was conducted in-person by a physician at the CIDEIM clinic. The therapeutic response was evaluated at week 13 as failure, improvement, or apparent cure, and at week 26 as cure or failure [17].

In the intervention (app) arm, it was not possible to evaluate the induration of lesions via photographs, only their epithelization, but induration is not considered an absolute criterion of cure [20].

In case of treatment failure, standard procedures based on national guidelines were followed for the administration of a second round of treatment with a different recommended drug. The responses to retreatment were outside of the study protocol and hence not reported here.

### Data management and statistical analysis

To generate an analysis dataset, the information on each patient obtained from the app, and from the electronic medical record routinely used in the CIDEIM, were collated into a CRF.

The statistical analysis followed the methods pre-defined in the protocol and in the statistical analysis plan, and was by intention to treat. For each endpoint, the comparison of main interest was between the two randomized arms. However, the same methods were used for the non-randomized control group. The primary endpoint, the proportion of participants monitored at target study week 26, was compared between the arms using a χ^2^ test. The *t* test was used to compare adherence in terms of the percentage of the prescribed doses taken by each patient. If an *F* test indicated that the variances differed between groups for the *t* test, then the Satterthwaite’s version was used [21]. As a post-hoc analysis, the Kaplan-Meier method was used to estimate the proportion of participants continuing in the study, in terms of the target study week of their last follow-up. No interim analysis was planned or carried out.

## Results

**Fig 3:**
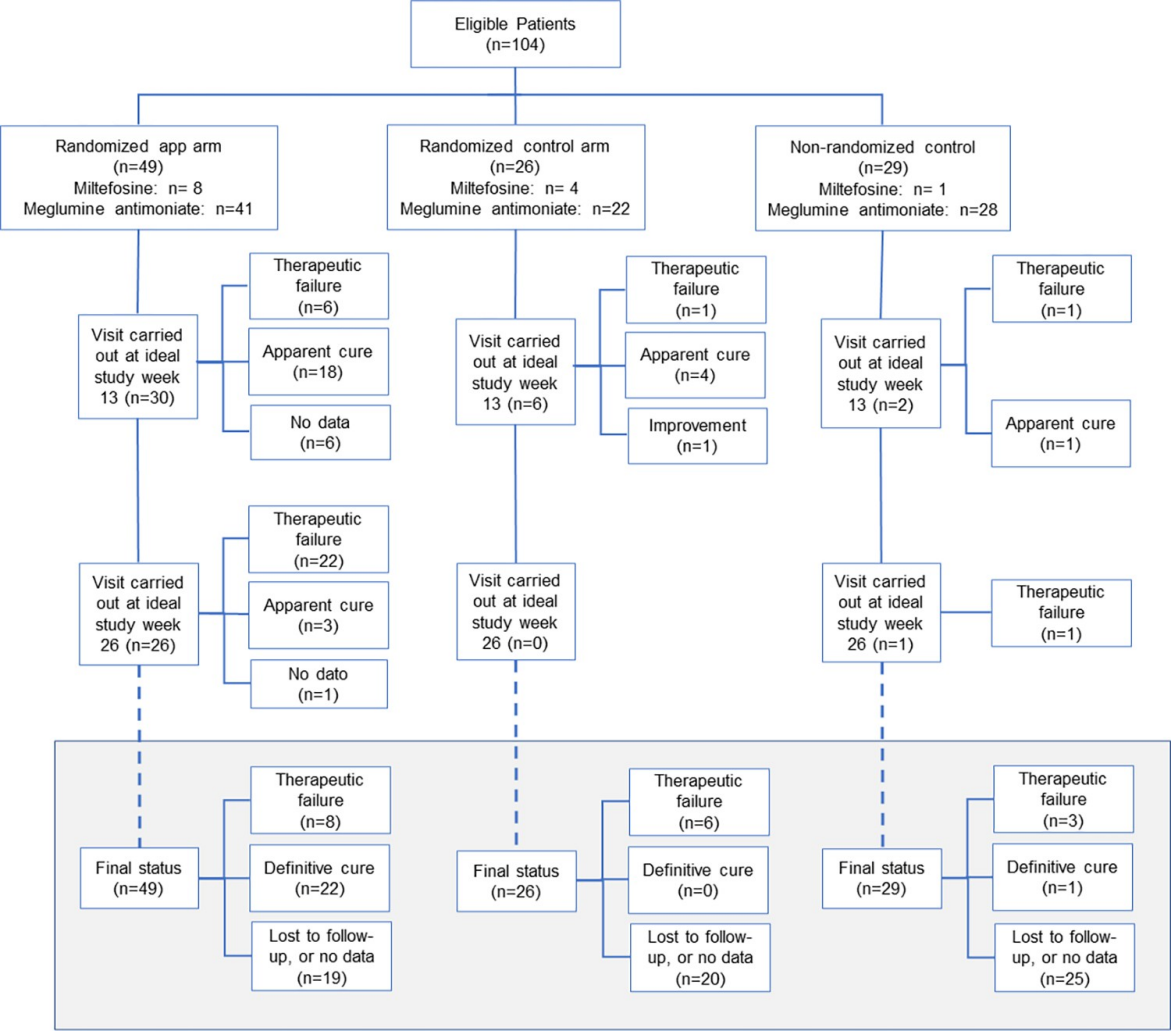
CONSORT flowchart. The lower part, in grey, is a summary of the outcomes, based on all the timepoints.

Baseline characteristics of the study participants are shown in Table 1, and the flowchart in Fig 3. We recruited a diverse population including Afrocolombian, *mestizo* and indigenous patient participants. The study was closed in December 2017, when the target total sample size of the randomized arms had been reached. The three study groups were similar in that the majority of patients were male and Afrocolombian, with median age between 20 and 30 years. More than 80% of each group were prescribed meglumine antimoniate, and the average number of ampoules administered, as a percentage of the number prescribed, was 88% or higher in each of the three groups. There was no statistically significant difference in the compliance with the prescribed treatment between the randomized arms (Table 2).

**Table 2:** Clinical status at end of treatment, and adherence with prescribed dose.

	App intervention (n = 49)	Randomized control (n = 26)	Non-randomized control (n = 29)
**Treatment prescribed**: n (%)			
Meglumine antimoniate	41 (83.7)	22 (84.6)	28 (96.6)
Miltefosine	8 (16.3)	4 (15.4)	1 (3.4)
**Among** meglumine antimoniate **patients, numbers having dose data available**: n (%)	33 (67.35)	11 (50.00)	10 (34.48)
**For those with data available, ampoules administered as a percentage of those prescribed**: median (range)	100 (25–100)	100 (33.3–100)	100 (100–105)
Mean (SD)	91.01 (22.13)	88.48 (23.53)	100.5 (1.58)^(a)^
Difference in means relative to the app arm (standard error)	-	-2.53 (7.82)	9.49 (3.89)
(95% confidence interval)	-	(-18.32; 13.27)	(1.59; 17.39)
p value (two sided)	-	0.749^(b)^	0.02^(c)^

^(a)^ 1 patient was administered more than the prescribed dose (84/80 ampoules).

^(b)^ *t* test, equal variances

^(c)^ *t* test, unequal variances

There were no serious adverse events, and no events of severe intensity. Among those receiving meglumine antimoniate, the proportion of participants with at least one adverse event was 78% (32/41) in the app arm, and 100% in the other two arms (22 in the randomized control arm and 28 in the non-randomized control group). For miltefosine, the corresponding proportions were 62.5% (5/8) in the app arm, and again 100% (out of 4 and 1, respectively, in the randomized control arm and the non-randomized control group). The median number of adverse events per person was 1 in each arm and under each treatment.

**Fig 4:**
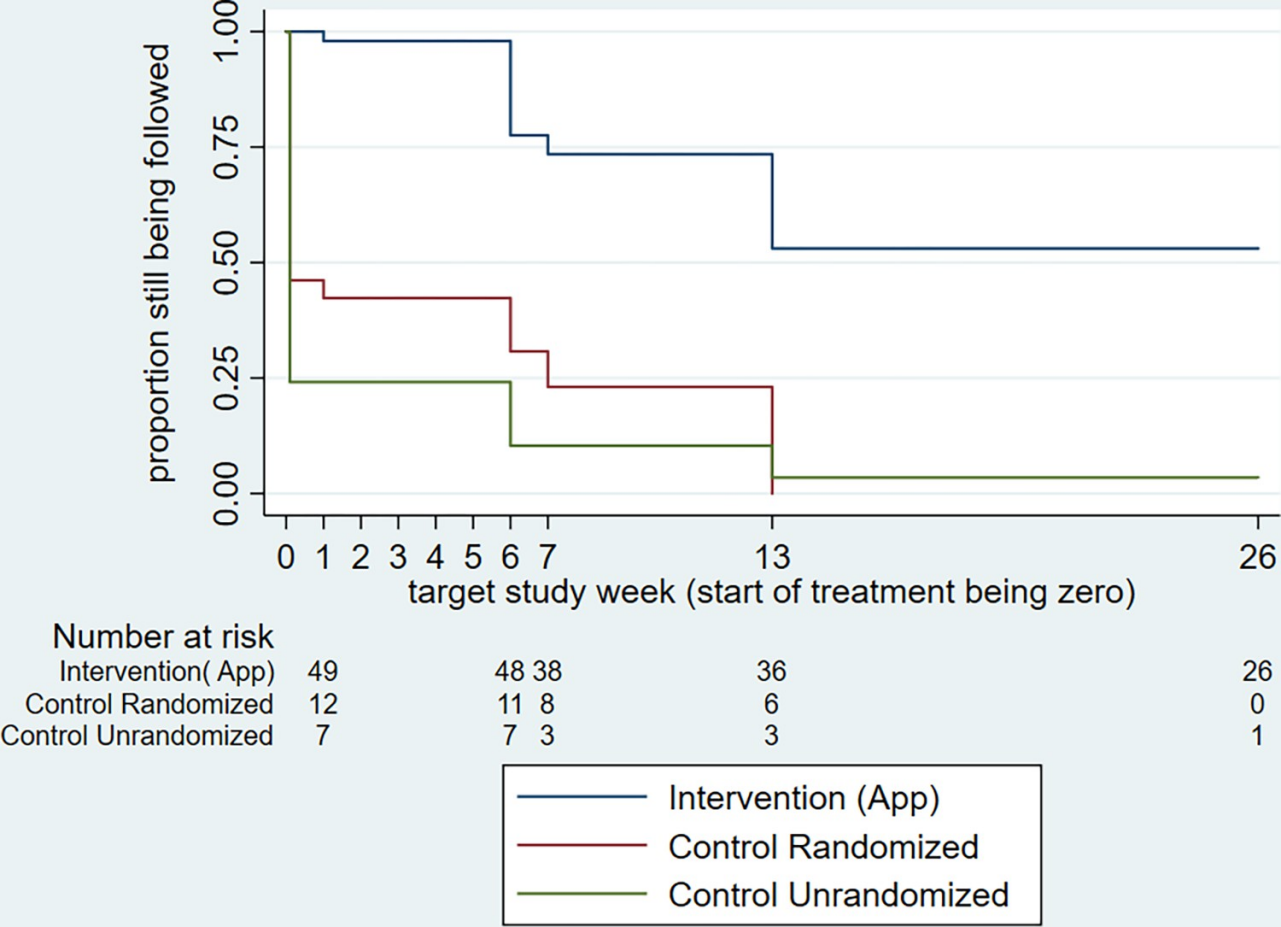
Kaplan-Meier survival. Proportion of patients who were followed over time in each group, showing that more app users were followed up at week 26.

**Fig 5:**
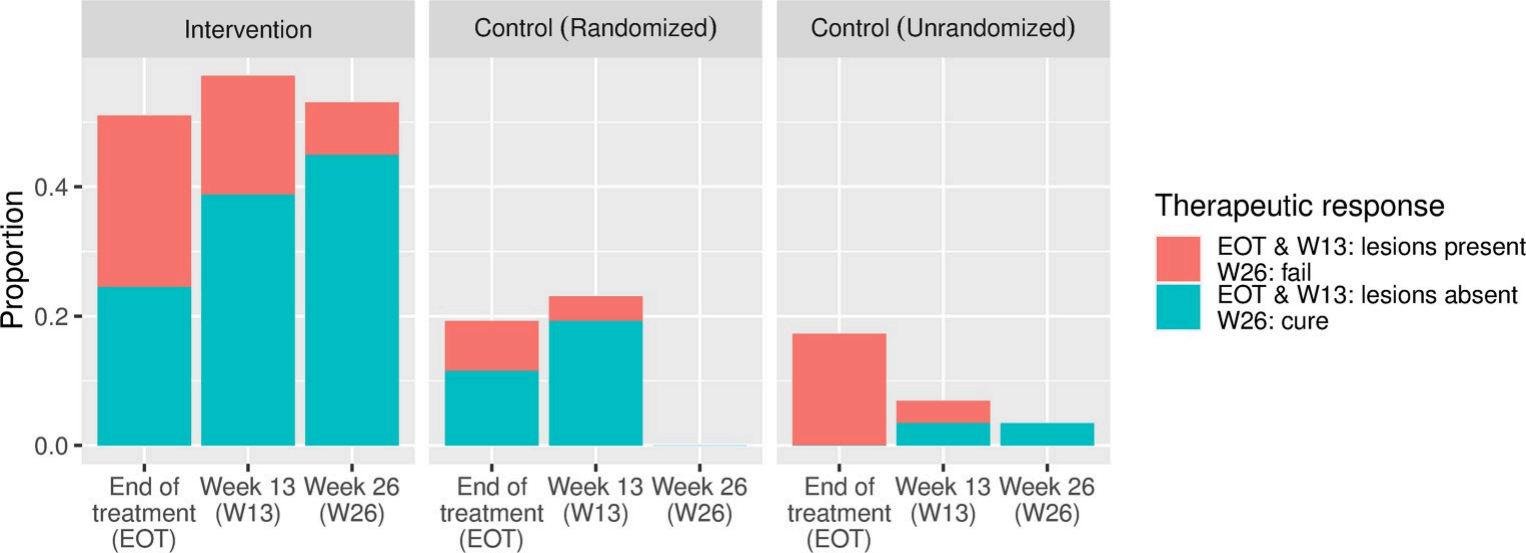
Proportion of patients in each group who were followed over time and had a favorable (green) or unfavorable (red) therapeutic response. At target study week 26, this means treatment cure or failure. Before week 26 it means absence or presence of lesions. In the app arm approximately half of the participants have the information, while, in the control groups, only one person has the information at week 26.

Of the 75 participants in the two randomized arms, 74 had information on whether or not they were followed up at target study week 26 (the primary endpoint of the trial). Among these, 26/49 (53.1%) were evaluated by active follow-up at this time in the intervention arm, and none (0/25, 0%) of those in the randomized control arm who, according to the standard-of-care were instructed to return for evaluation at week 13 and 26 (difference = 53.1%, 95% confidence interval 39.1–67.0%, p<0.001). This difference is also evident at target study week 26 in the Kaplan-Meier plot (Fig 4), as are differences of similar magnitude throughout the post-treatment period, at weeks 7 and 13. Clinical progression is shown in Fig 5, showing that of the 26 participants followed up at target study week 26 in the intervention arm, 22 (84.6%) were cured, establishing the effectiveness of the treatments received. Across the other two groups (randomized control and non-randomized control), only one person was evaluated at target study week 26, and was also cured. This confirms the lack of information on progression of lesions (effectiveness of treatment) through the standard institution-based passive monitoring of treatment (i.e. standard of care). The virtual absence of information on response to treatment for the two control groups after completion of treatment, precluded evaluation of the influence of active follow-up monitoring on management of adverse events or the outcome of treatment.

## Discussion

This study established the functionality of the Guaral +ST app for monitoring of treatment under the conditions evaluated: use by CHV in rural settings with poor access to mobile networks. This approach to active monitoring yielded a significant gain in information on adverse events, adherence to, and outcome of treatment, supporting the feasibility of active monitoring of treatment and outcome by CHV using this mHealth app. CHV, particularly those who were members of the civil defense and had permits and a distinguishing uniform, were able to move more freely, providing outreach to dispersed communities including some affected by the resurgence of violence. Nevertheless, insecurity did constrain the functional range of the CHV and the proportion of patients that were evaluated or lost to follow-up. Even in the intervention arm, 46.9% had been lost to follow-up by week 26, reflecting difficulty in mobility in the study area for geographical and socioeconomic reasons. However, in the intervention arm, retention was notably greater (approximately 75%) at week 13, and this time point is considered an acceptable time to define outcome [20].

This RCT experience evaluating mHealth underscored the importance of contextual awareness and engagement and articulation of CHVs with peripheral health facilities and other entities working with the communities such as civil defense, Red Cross, Doctors Without Borders, and community councils. These organizations and their personnel provided a network of communication and support in locating and monitoring individuals with CL.

Connectivity presented challenges to the use of the Guaral +ST app and has been reported and recognized as a potential barrier for other mHealth strategies in dispersed rural communities where internet coverage is patchy and unstable [7]. In this study, CHV were able to record information on adherence to treatment, adverse reactions and therapeutic outcome without connectivity, and transmit the saved data afterwards when in an area having internet coverage using an asynchronous “store-and-forward” strategy. The limitation of connectivity could therefore be managed, though not totally overcome, in the setting of this study. Another potential limitation of the study is that a non-randomized control group was added, and comparisons with this group are restricted in terms of internal validity. On the other hand, this group was included because of another limitation, namely that the intervention could only be carried out where a CHV was available and security concerns did not impede interaction with the dispersed households, a factor that restricts the external validity (generalizability). Another potential limitation is that the intervention arm was evaluated via photographs, while the control arm was evaluated in person. However, we have recently shown that evaluation by photographs is highly accurate [12].

Based on this and prior experience with mHealth for community empowerment [5, 6, 22], receptivity of the CHVs to learning to use the mobile app and aptitude for digital technology was an enabling factor and is an important consideration in identifying CHVs for the introduction of mHealth approaches to increase access to medical attention. The distribution of CHVs having the skill set and safety profile to reach and monitor individuals with CL residing in dispersed and culturally diverse communities is uneven. Consequently, innovative strategies are needed to increase the number, geographic distribution and competencies of CHVs to adopt mobile technologies.

The people-centered design of the Guaral +ST app included embedded instructions and educational components [6]. In addition to adjusting the app in response to the outcome of this initial evaluation, the instructional feature could be utilized to address frequently arising questions or difficulties. This app was intended as a data collection and transfer tool as well as a monitoring and empowering strategy for CHV. Complexity of data management is a possible limitation of mHealth interventions [23] and, in our case, generation of a final analysis dataset from the app and standard data sources required collation into a CRF. In design terms, the people-centered approach was implemented with greater focus on the app users, and therefore the patient-CHW interface, for example. However, generation of an analysis dataset for the use of the researchers proved to be challenging. Going forward, the app could be simplified to be tool for monitoring with minimal database architecture and management requirements. For adverse events, such a simplified app would provide guidance for the management and alleviation of mild and moderate drug reactions. The simplification would also facilitate implementation, adoption and scale-up to other communities both in terms of the skills, connectivity challenges, reporting through the health system and reduced requirements for data transfer and long-term storage.

The combination of mHealth and empowerment of CHV through training to effectively utilize the app, responded to an unmet patient need and provided previously unavailable information to the health system on the effectiveness of the treatments administered as standard of care. This approach shifts responsibility for active monitoring of patients during treatment to communities in alliance with health professionals and institutions, similar to the ECHO model which is effective for hepatitis C [24]. Such an approach could be expanded to other transmissible diseases in an integrated control strategy for diseases affecting rural communities including TB and malaria, to both leverage and strengthen active monitoring programs such as DOTs [25].

In conclusion, this study provides proof of concept for mHealth monitoring of CL treatment in remote and complex settings by community health volunteers, deliver of improved care, and provision of previously unavailable information to the health system on effectiveness of treatment.

## Supporting information

S1 Table Study procedures. (DOCX)

S2 Table CONSORT checklist. (DOC)
