# Supplementary material for: Randomized trial evaluating an mHealth intervention for the early community-based detection and follow-up of cutaneous leishmaniasis in rural Colombia
Source: PLoS Negl Trop Dis. 2023 Mar 27;17(3):e0011180. doi: 10.1371/journal.pntd.0011180 (PMC10079216; doi:10.1371/journal.pntd.0011180)
Supplement: S1 Table — (DOCX) [file pntd.0011180.s001.docx]

| Supplemental Table: study procedures. | | | | | | | | | | |
| --- | --- | --- | --- | --- | --- | --- | --- | --- | --- | --- |
| Procedure | Arm / group | Site | Screening & recruitment | Randomization | Treatment | End of treatment | Week 7 | Week 13 | Week 26 |  |
| Informed consent for CPR evaluation | All | CIDEIM-Tumaco | X |  |  |  |  |  |  |  |
| CPR evaluation with mobile application. | All | CIDEIM-Tumaco | X |  |  |  |  |  |  |  |
| Evaluation of eligibility criteria | All | CIDEIM-Tumaco | X |  |  |  |  |  |  |  |
| Parasitological confirmation | All | CIDEIM-Tumaco | X |  |  |  |  |  |  |  |
| Medical history and initial physical exam | All | CIDEIM-Tumaco | X | X |  |  |  |  |  |  |
| Informed consent for participation in study proper | All | CIDEIM-Tumaco | X |  |  |  |  |  |  |  |
| Taking initial photographs | All | CIDEIM-Tumaco | X |  |  |  |  |  |  |  |
| Treatment indication and formulation | All | CIDEIM-Tumaco |  | X |  |  |  |  |  |  |
| Randomization | The two randomized arms | CIDEIM-Tumaco |  | X |  |  |  |  |  |  |
| Treatment administration | All | CIDEIM-Tumaco or place of residence |  |  | X |  |  |  |  |  |
| Evaluation of adverse events  Via the Guaral +ST mobile app and/or a paper form. | App arm | Place of residence |  |  | X | X |  |  |  |  |
| Via appointment with medical personnel, entered into the electronic medical record (possibly via a paper form). | Control (randomized or not) | CIDEIM-Tumaco or Health post |  |  |  | X |  |  |  |  |
| Therapeutic response follow-up:  Via the (Guaral +ST) mobile app and/or a paper form. | App arm | Place of residence |  |  |  |  | X | X | X |  |
| In electronic medical record (possibly via a paper form) | Control (randomized or not) | CIDEIM-Tumaco |  |  |  |  | X | X | X |  |
| Information synchronization between mobile devices and SND | App arm |  | X | X | X | X | X | X | X |  |
