# Supplementary material for: Randomized trial evaluating an mHealth intervention for the early community-based detection and follow-up of cutaneous leishmaniasis in rural Colombia
Source: PLoS Negl Trop Dis. 2023 Mar 27;17(3):e0011180. doi: 10.1371/journal.pntd.0011180 (PMC10079216; doi:10.1371/journal.pntd.0011180)
Supplement: S2 Table — (DOC) [file pntd.0011180.s002.doc]

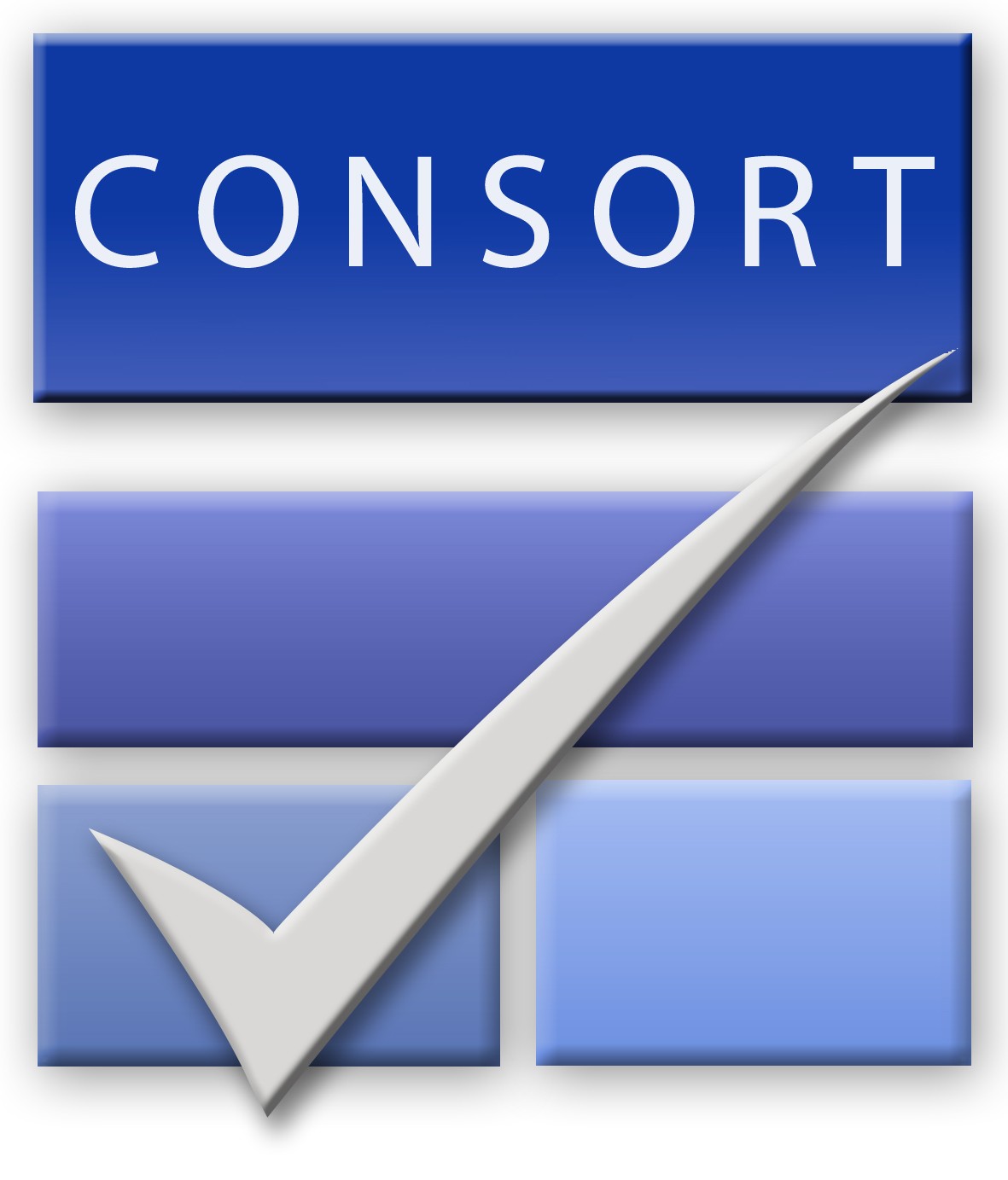
CONSORT 2010 checklist of information to include when reporting a randomised trial*

| Section/Topic | Item No | Checklist item | Reported on page No |
| --- | --- | --- | --- |
| Title and abstract | | | |
|  | 1a | Identification as a randomised trial in the title | In the title (p1) |
|  | 1b | Structured summary of trial design, methods, results, and conclusions (for specific guidance see CONSORT for abstracts) | In the abstract (p1) |
| Introduction | | | |
| Background and objectives | 2a | Scientific background and explanation of rationale | “Introduction” section |
|  | 2b | Specific objectives or hypotheses | “Introduction” section |
| Methods | | | |
| Trial design | 3a | Description of trial design (such as parallel, factorial) including allocation ratio | “Design of the mHealth app and of the randomized trial” subsection of the methods. |
|  | 3b | Important changes to methods after trial commencement (such as eligibility criteria), with reasons | “Non-randomized control group” subsection of the methods. |
| Participants | 4a | Eligibility criteria for participants | “Inclusion and exclusion criteria” subsection of the methods. |
|  | 4b | Settings and locations where the data were collected | “Setting” subsection of the methods. |
| Interventions | 5 | The interventions for each group with sufficient details to allow replication, including how and when they were actually administered | The following subsections of the methods: “Follow-up during treatment”, “Endpoints”, “Adverse events”, “Therapeutic response”. |
| Outcomes | 6a | Completely defined pre-specified primary and secondary outcome measures, including how and when they were assessed | “Endpoints” subsection of the methods. |
|  | 6b | Any changes to trial outcomes after the trial commenced, with reasons | (none) |
| Sample size | 7a | How sample size was determined | “Sample size” subsection of methods. |
|  | 7b | When applicable, explanation of any interim analyses and stopping guidelines | None. This is stated in the “Data management and statistical analysis” subsection of the methods. |
| Randomisation: |  |  |  |
| Sequence generation | 8a | Method used to generate the random allocation sequence | “Randomization” subsection of methods. |
|  | 8b | Type of randomisation; details of any restriction (such as blocking and block size) | “Randomization” subsection of methods. |
| Allocation concealment mechanism | 9 | Mechanism used to implement the random allocation sequence (such as sequentially numbered containers), describing any steps taken to conceal the sequence until interventions were assigned | “Randomization” subsection of methods. |
| Implementation | 10 | Who generated the random allocation sequence, who enrolled participants, and who assigned participants to interventions | “Randomization” subsection of methods. |
| Blinding | 11a | If done, who was blinded after assignment to interventions (for example, participants, care providers, those assessing outcomes) and how | “Randomization” subsection of methods. |
|  | 11b | If relevant, description of the similarity of interventions | Not relevant due to lack of blindness. |
| Statistical methods | 12a | Statistical methods used to compare groups for primary and secondary outcomes | “Data management and statistical analysis” subsection of methods. |
|  | 12b | Methods for additional analyses, such as subgroup analyses and adjusted analyses | “Data management and statistical analysis” subsection of methods. |
| Results | | | |
| Participant flow (a diagram is strongly recommended) | 13a | For each group, the numbers of participants who were randomly assigned, received intended treatment, and were analysed for the primary outcome | Flowchart figure. |
|  | 13b | For each group, losses and exclusions after randomisation, together with reasons | Flowchart figure. |
| Recruitment | 14a | Dates defining the periods of recruitment and follow-up | First paragraph of Results section. |
|  | 14b | Why the trial ended or was stopped | First paragraph of Results section. |
| Baseline data | 15 | A table showing baseline demographic and clinical characteristics for each group | Table 1. |
| Numbers analysed | 16 | For each group, number of participants (denominator) included in each analysis and whether the analysis was by original assigned groups | Flowchart figure, Table 2, third paragraph of results section, “Data management and statistical analysis” subsection of methods. |
| Outcomes and estimation | 17a | For each primary and secondary outcome, results for each group, and the estimated effect size and its precision (such as 95% confidence interval) | Table 2, third paragraph of results section. |
|  | 17b | For binary outcomes, presentation of both absolute and relative effect sizes is recommended | We have presented only absolute effect sizes. |
| Ancillary analyses | 18 | Results of any other analyses performed, including subgroup analyses and adjusted analyses, distinguishing pre-specified from exploratory | Third paragraph of results section, “Data management and statistical analysis” subsection of methods. |
| Harms | 19 | All important harms or unintended effects in each group (for specific guidance see CONSORT for harms) | Second paragraph of results section. |
| Discussion | | | |
| Limitations | 20 | Trial limitations, addressing sources of potential bias, imprecision, and, if relevant, multiplicity of analyses | Third paragraph of discussion section. |
| Generalisability | 21 | Generalisability (external validity, applicability) of the trial findings | Third paragraph of discussion section. |
| Interpretation | 22 | Interpretation consistent with results, balancing benefits and harms, and considering other relevant evidence | Final paragraph of discussion section. |
| Other information | | |  |
| Registration | 23 | Registration number and name of trial registry | “Design of the mHealth app and of the randomized trial” subsection of the methods: ISRCTN54865992. |
| Protocol | 24 | Where the full trial protocol can be accessed, if available | “Design of the mHealth app and of the randomized trial” subsection of the methods. |
| Funding | 25 | Sources of funding and other support (such as supply of drugs), role of funders | This is specified in the journal’s online submission system. |

*We strongly recommend reading this statement in conjunction with the CONSORT 2010 Explanation and Elaboration for important clarifications on all the items. If relevant, we also recommend reading CONSORT extensions for cluster randomised trials, non-inferiority and equivalence trials, non-pharmacological treatments, herbal interventions, and pragmatic trials. Additional extensions are forthcoming: for those and for up to date references relevant to this checklist, see [www.consort-statement.org](http://www.consort-statement.org/).
